# Attenuated *Salmonella* typhimurium delivering DNA vaccine encoding duck enteritis virus *UL24* induced systemic and mucosal immune responses and conferred good protection against challenge

**DOI:** 10.1186/1297-9716-43-56

**Published:** 2012-07-06

**Authors:** Xia Yu, Renyong Jia, Juan Huang, Bin Shu, Dekang Zhu, Qing Liu, Xinghong Gao, Meng Lin, Zhongqiong Yin, Mingshu Wang, Shun Chen, Yin Wang, Xiaoyue Chen, Anchun Cheng

**Affiliations:** 1 Institute of Preventive Veterinary Medicine, Sichuan Agricultural University, Chengdu, Sichuan, 611130, People's Republic of China; 2 Avian Disease Research Center, College of Veterinary Medicine of Sichuan Agricultural University, 46 Xinkang Road, Ya’an, Sichuan, 625014, People's Republic of China; 3 Key Laboratory of Animal Disease and Human Health of Sichuan Province, Sichuan Agricultural University, Chengdu, Sichuan, 611130, People's Republic of China

## Abstract

Orally delivered DNA vaccines against duck enteritis virus (DEV) were developed using live attenuated *Salmonella* typhimurium (SL7207) as a carrier and *Escherichia coli* heat labile enterotoxin B subunit (LTB) as a mucosal adjuvant. DNA vaccine plasmids pVAX-UL24 and pVAX-LTB-UL24 were constructed and transformed into attenuated *Salmonella* typhimurium SL7207 resulting SL7207 (pVAX-UL24) and SL7207 (pVAX-LTB-UL24) respectively. After ducklings were orally inoculated with SL7207 (pVAX-UL24) or SL7207 (pVAX-LTB-UL24), the anti-DEV mucosal and systemic immune responses were recorded. To identify the optimum dose that confers maximum protection, we used different doses of the candidate vaccine SL7207 (pVAX-LTB-UL24) during oral immunization. The strongest mucosal and systemic immune responses developed in the SL7207 (pVAX-LTB-UL24) (10^11^ CFU) immunized group. Accordingly, oral immunization of ducklings with SL7207 (pVAX-LTB-UL24) showed superior efficacy of protection (60-80%) against a lethal DEV challenge (1000 LD_50_), compared with the limited survival rate (40%) of ducklings immunized with SL7207 (pVAX-UL24). Our study suggests that the SL7207 (pVAX-LTB-UL24) can be a candidate DEV vaccine.

## Introduction

Duck viral enteritis (DVE, also called duck plague), caused by Anatid herpesvirus 1 (Duck enteritis virus, DEV), is an acute, contagious viral disease of ducks, geese and swans, accounting for a high mortality rate in ducks and decreased egg production, leading to heavy economic losses [1-4]. The symptoms of this disease include vascular damage, eruptions at specific locations on the mucosal surface of the gastrointestinal tract, lesions of lymphoid organs and degenerative sequelae in parenchymatous organs [5].

Immunization of ducks is an efficient way to prevent DEV infection [6,7]. The commonly used DEV attenuated live vaccine, provides a good protection against DEV infection [8]. However, the production and supply of the vaccine is insufficient, considering the large number of domestic and wild ducks [6]. Additionally, sometimes this vaccine fails to protect ducks after intramuscular or subcutaneous vaccination and, because it is grown in chick embryos, it may harbor other infectious agents such as H5N1 [6,9]. Therefore, a novel and more effective vaccine to protect against DEV infection is urgently required. Recently, some enteropathogenic bacteria [10] have been used as effective carriers for DNA vaccine including attenuated strains of *Listeria monocytogenes*[11], *Salmonella* spp [12] and *Shigella* spp [13]. These bacteria are attractive vectors to deliver DNA vaccines to immunological inductive sites at mucosal surfaces and antigen-presenting cells (APC), which can improve mucosal and systemic responses against pathogens [14,15]. Amongst these bacteria, attenuated *Salmonella* has been extensively studied [15,16]. However, the use of attenuated *Salmonella* typhimurium as a DNA vaccine carrier in DEV has not yet been reported. A few antigens derived from pathogenic microorganisms, such as *Escherichia coli* heat-labile enterotoxin (LT), can be used as adjuvants to improve systemic and mucosal responses [17]. The nontoxic B subunit (LTB) is commonly used for this purpose [18,19]. These strategies of DNA vaccine combined with adjuvant might provide new opportunities in the development of DEV vaccine.

DEV was classified as a separate genus of the *Alphaherpesvirinae* subfamily in the Herpesviridae family [1,2]. One gene *UL24*, is considered to be a core herpesvirus gene and is conserved among most of the herpes viruses [20,21], and null mutations or mutations in the conversed regions of *UL24* can confer a syncytial phenotype and result in decreased viral yields in cultured cells, indicating that *UL24* is important for efficient viral replication [22,23]. In addition, *UL24* protein has the ability to elicit a specific antibody response [24].

In this study, we used LTB as an adjuvant fused to *UL24* gene and the attenuated *S.* typhimurium aroA^-^ strain SL7207 as a vector to deliver DEV DNA vaccines. The results indicate that oral immunization of the recombinant *S.* typhimurium could induce specific immune response against DEV.

## Materials and methods

### Bacterial strains, plasmids, experimental ducklings

Eukaryotic expression pVAX1 (Invitrogen, Carlsbad, California, USA), which contains the cytomegalovirus (CMV) promoter and bovine growth hormone (BGH) poly A signal, and enterotoxigenic *E. coli* K88ac were generously provided by Professor Sanjie Cao of Sichuan Agricultural University, China. The attenuated *S.* typhimurium aroA^-^ strain SL7207 (*S*. typhimurium 2337–65 derivative *hisG46*, DEL407 [*aroA*::Tn*10* (Tc^s^)]) was kindly provided by Professor Kai Schulze of Helmholtz Centre for Infection Research (Germany). 7-day-old Tianfu ducklings were purchased from commercial duck farms (Ya’an, China) and fed under standard conditions.

### Construction of expression plasmids

**Figure 1:**
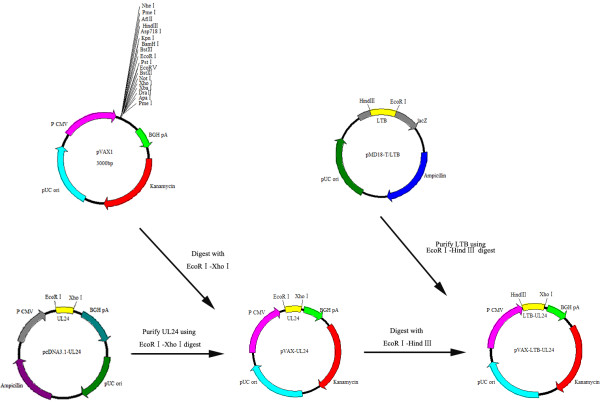
**A schematic diagram of the construction of pVAX-UL24 and pVAX-LTB-UL24.** A schematic diagram of UL24 gene cloned into the pVAX-1 vector and a schematic diagram of LTB gene cloned into the recombinant plasmid pVAX-UL24.

*E. coli* K88ac genomic DNA was extracted using cetyl trimethylammonium bromide (CTAB) [25]. According to the DNA sequence of LTB published in GenBank (Accession:EU113245.1), the primers were designed (p1: 5’- TTAAACTTAAGCTTATGAATAAAGTAAAATGT-3’, p2: 5’-GAATTCGTTTTCCATACTGATTGC-3’) to amplify the LTB gene containing a *Hin*dIII site (underlined) in primer 1 and an *EcoRI* site (underlined) in primer 2. The amplified DNA fragment of the *LTB* gene was cloned into a pMD-T18 vector (TaKaRa, Dalian, Shangdong, China) and sequenced. The 1230 bp *UL24* gene fragment was cut from the pcDNA3.1 plasmid (lab collection) by *Eco*RI and *Xho*І digestion and inserted into the pVAX1 expression vector, resulting in pVAX-UL24 (Figure 1). Fragments of *Hind*III- and *Eco*RІ-digested pMD-T18/LTB were inserted into the *Hin*dIII/*Eco*RІ site of pVAX-UL24 (Figure 1). The recombinant pVAX-UL24 and pVAX-LTB-UL24 were confirmed by restriction enzyme digestion and sequencing.

### Transient expression of the recombinant plasmids

When COS-7 cells growing in 6-well plates (Corning Life Sciences, Corning, New York, USA) were 60-90% confluent, the cells were transiently transfected with plasmids pVAX-UL24, pVAX-LTB-UL24 and pVAX1 using Lipofectamine 2000 (Invitrogen), respectively. Forty-eight hours after transfection, the cells were washed with phosphate-buffered-saline (PBS) and were fixed with 4% paraformaldehyde for 15 min at room temperature and washed again. After being permeabilized with 0.2% Triton X-100 (v/v in PBS) for 10 min, the cells were blocked in 5% BSA for 1 h at 37 °C. Then, the samples were incubated with diluted primary and secondary antibodies at 37 °C for 1 h, respectively. Primary antibodies used were purified rabbit anti-DEV UL24 polyclonal and secondary antibodies were fluorescein isothiocyanate (FITC)-conjugated goat anti-rabbit IgG (Sigma, St. Louis, Missouri, USA). After the cell nuclei were counterstained with 4',6-diamidino-2-phenylindole (DAPI) for 10 min at room temperature, the coverslips were mounted onto glass slides with a drop of buffered glycerol and analyzed by fluorescence microscopy (Nikon, Tokyo, Japan).

### Transformation of attenuated S. typhimurium strain SL7207 with DNA vaccine plasmids

The recombinant plasmids pVAX-UL24, pVAX-LTB-UL24 and control vector pVAX1 were transformed into attenuated *S.* typhimurium strain SL7207 by electroporation using the Micropulser Electroporator (Bio-Rad, Hercules, California, USA). The positive transformants were selected on LB agar containing kanamycin (50 μg/mL) and then were confirmed by sequencing and PCR. The constructs were named strain SL7207 (pVAX-UL24), strain SL7207 (pVAX-LTB-UL24) and strain SL7207 (pVAX1) respectively. The primers of *UL24* gene were designed as follows: primer 3: 5'-ATGGCATCGAAGGTACAGAAA AAGC-3' (forward) and primer 4: 5'-CTCGAGCTAGTGTTTAGTGGTCTGAA-3' (reverse). The primers to amplify *LTB-UL24* (about 1600 bp in length) were primer 1 (as aforementioned) and primer 4.

### RT-PCR detection of transcripts in vivo

2-week-old ducklings were orally administered 1 × 10^10^ CFU of SL7207 (pVAX-UL24) or SL7207 (pVAX-LTB-UL24), and control ducklings were given the same dose of SL7207 (pVAX1). Three days after the immunization, ileums (from three ducklings immunized with SL7207 (pVAX-UL24), SL7207 (pVAX-LTB-UL24) or SL7207 (pVAX1)) were removed and pooled. The *UL24* and *LTB-UL24* transcripts (mRNA) were then analyzed by reverse transcriptase-polymerase chain reaction (RT-PCR).

### Immunization and serum sampling

Ducklings, randomly divided into 6 groups (40 per group), were allowed to adapt to the new environment for 7 days, deprived of food and water for 4 h prior to immunization, and immunized three times at 7-day intervals. For oral immunization, each duckling received 100 μL of 10% NaHCO_3_ intragastrically 30 min before immunization to neutralize gastric acids. Ducklings of group A, B and C were intragastrically inoculated with SL7207 (pVAX-LTB-UL24) at doses of 10^11^, 10^10^ and 10^9^ CFU per duckling respectively. Ducklings in groups D and E were inoculated intragastrically with SL7207 (pVAX-UL24) and the control strain SL7207 (pVAX1), respectively, at 10^10^ CFU per duckling. Group F ducklings received PBS and were used as a negative control.

At weeks 1, 2, 3, 4, 5, 6 and 8 after the first immunization, three ducklings of each group were sacrificed for sera, bile and duodenum. To prepare duodenal fluid, its connective and fat tissues in serosa were removed in PBS and were opened to expose their lumens. After a 1 cm section of this intestinal tract was placed in 1 mL of PBS containing 100 μg/mL of trypsin inhibitor (Sigma), the mixture was centrifuged and the supernatant was collected. Accordingly, the bile obtained was centrifuged and the supernatant was collected. All of these samples were stored at −20 °C prior to analysis.

### Measurement of antibody levels

Enzyme-linked immunosorbent assay (ELISA) was used to analyze DEV-specific antibodies in serum, duodenum and bile. In brief, 96-well polystyrene microtitre plates were coated with 100 μL 0.25 μg/mL purified DEV-antigen and incubated overnight at 4 °C. The plates were washed three times with PBS containing 0.05% Tween 20 (PBST) and blocked by incubation with 100 μL of blocking solution (1% BSA in PBST) for 1 h at 37 °C. After washing, a 100-μL volume of diluted duckling serum was added to each well, and incubated for 1 h at 37 °C. Rabbit anti-duck IgG or IgA-horseradish peroxidase conjugate (Sigma) was used as the secondary antibody at 1:2000 dilutions and incubated for 1 h at 37 °C, then washed again. The substrate, 3,3’,5,5’-tetramethy1 benzidine (TMB), added and incubated for 30 min at 37 °C. 50 μL of 2 mol/L H_2_SO_4_ was added to stop the reaction, and then the optical density (OD) at 450 nm was measured using an ELISA reader.

### Measurement of neutralizing antibody responses

Serum samples were inactivated at 56 °C for 30 min and diluted in serial twofold dilutions in MEM (GIBCO, Grand Island, New York, USA). Each sample was mixed with an equal volume containing 200 TCID_50_ of DEV and incubated for 1 h at 37 °C. One-hundred microliters of the above serum-virus mixture was transferred to duck embryo fibroblast monolayers on 96-well culture plates (Corning Life Sciences) and incubated at 37 °C for 1 h. The mixture in each well was then replaced with MEM containing 2% FCS (GIBCO). Cytopathic effects were observed for 4 days and the end-point dilution of each serum sample was calculated by the Reed-Muench formula [26].

### Detection of CD4+ and CD8+ T cells by flow cytometry

Peripheral blood lymphocytes (PBL) were isolated and indirect staining of the cells was carried out as described elsewhere [7]. Briefly, PBL were isolated from heparinized blood samples, washed twice by PBS, and adjusted to a final concentration of 5 × 10^5^ cells per mL. Then 1:200 diluted anti-duck CD4 monoclonal antibody and 1:1000 diluted anti-duck CD8 monoclonal antibody (AbD Serotec Ltd, Oxford, UK) were added into the cells. After incubation for 30 min at 4 °C in the dark, FITC-labeled goat anti-mouse IgG (AbD Serotec Ltd) was added. Subsequently, the cells were washed with PBS and resuspended in 500 μL PBS, followed by flow cytometric analysis. Viable lymphocytes were gated on the basis of forward and side scatter characteristics, and 10 000 events were analyzed for positive staining with FITC. Data analysis was carried out using BD FACSAria software.

### Protection studies

To assess the protection of DNA vaccines against DEV in immunized ducklings, 10 ducklings from each group were orally challenged with 1000 lethal doses, 50% (LD_50_) of DEV at 6 weeks after primary immunization. These ducklings were monitored daily for survival for 10 days after challenge.

## Results

### Construction and transient expression of pVAX-UL24 and pVAX-LTB-UL24 in COS-7 cells

**Figure 2:**
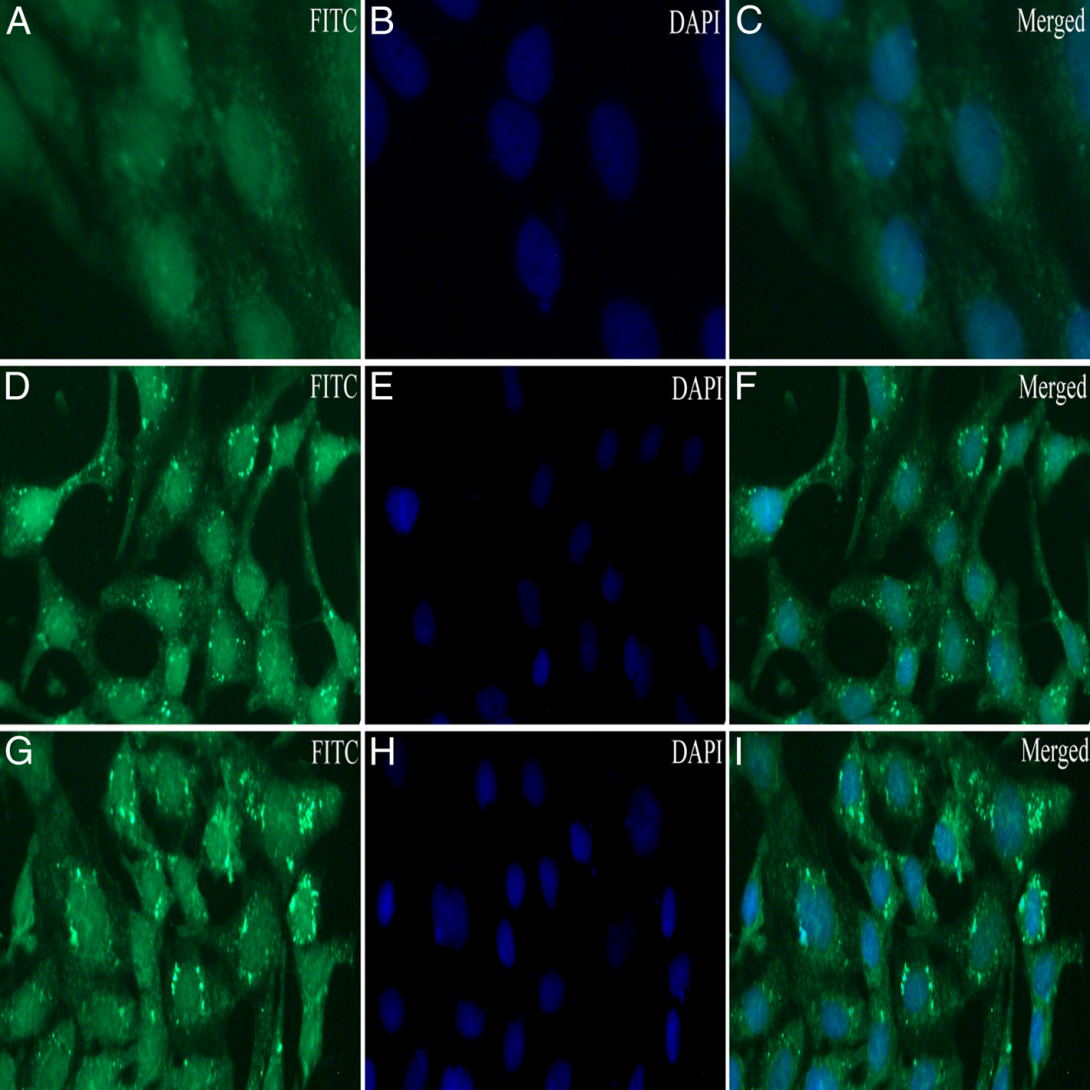
**Indirect immunofluorescence detection of the expression of pVAX-UL24 and pVAX-LTB-UL24 in COS-7 cells (× 200).A**, **D** and **G** show FITC staining; **B**, **E** and **H** show DAPI staining; **C**, **F** and **I** are the merged images of FITC and DAPI staining. (**A**, **B**, **C**) Indirect immunofluorescence result of COS-7 cells transfected with pVAX1; (**D**, **E**, **F**) Indirect immunofluorescence result of COS-7 cells transfected with pVAX-UL24; (**G**, **H**, **I**) Indirect immunofluorescence result of COS-7 cells transfected with pVAX-LTB-UL24.

Indirect immunofluorescence experiments demonstrated that specific fluorescent granules dispersed in the nucleus and cytoplasm in the cells transfected with pVAX-UL24 or pVAX-LTB-UL24 (Figure 2D-I), whereas no fluorescence was detected in cells infected with plasmid pVAX1 (Figure 2A-C), indicating that UL24 and LTB-UL24 were expressed in COS-7 cells. The recombinant plasmid pVAX-UL24 or pVAX-LTB-UL24 was successfully transformed into attenuated *S.* typhimurium and analyzed by PCR and then sequenced (data not shown).

### Transcripts of UL24 and LTB-UL24 genes in vivo

**Figure 3:**
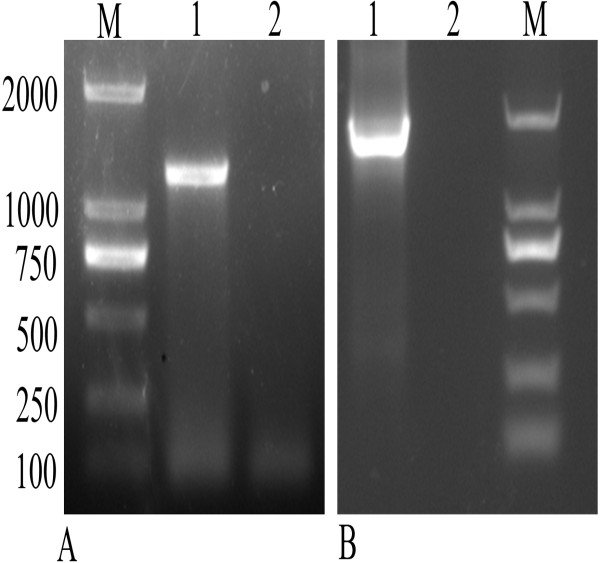
**RT-PCR detection of the transcripts of UL24 and LTB-UL24 genes in vivo.** (**A**) Lane M, DL 100–2000 marker; lane 1, ducklings were orally immunized with SL7207 (pVAX-UL24); lane 2, control ducklings were orally immunized with SL7207 (pVAX1); (**B**) lane 1, ducklings were orally immunized with SL7207 (pVAX-LTB-UL24); lane 2, control ducklings were orally immunized with SL7207 (pVAX1); lane 3, DL 100–2000 marker.

To test the expression of DEV DNA vaccines in vivo, total cellular RNA of ilea was isolated at day 3 after immunization, and then was analyzed by RT-PCR for the transcripts of *UL24* or *LTB-UL24* gene. As shown in Figure 3, a DNA fragment about 1230 bp was amplified from the RNA of ducklings immunized with SL7207 (pVAX-UL24), and a fragment about 1600 bp was amplified from ducklings immunized with SL7207 (pVAX-LTB-UL24). However, there were no DNA fragments amplified from RNA of control ducklings immunized with SL7207 (pVAX1). These data showed that UL24 and LTB-UL24 were transcribed from DNA to mRNA and therefore may be capable of expressing UL24 or LTB-UL24 in duckling cells in vivo.

### Humoral immune responses of ducklings

**Figure 4:**
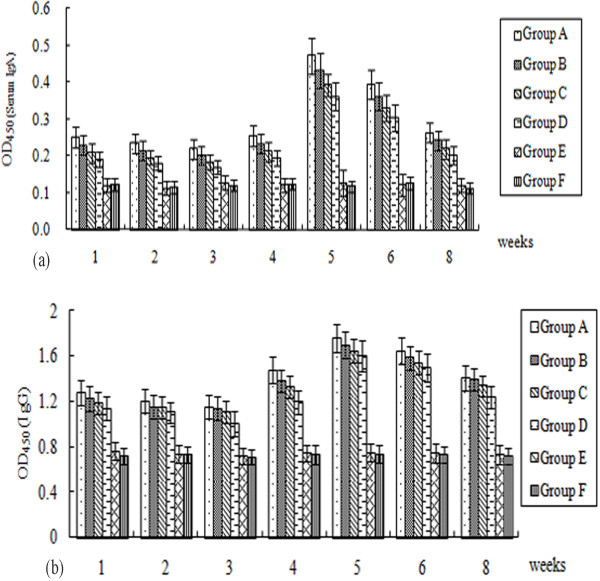
**Humoral immune responses to DEV measured by ELISA.** (**A**) Serum IgG responses to DEV. (**B**) Serum IgA responses to DEV. Sera were obtained at different time points and assayed by end-point dilution ELISA using DEV as the coating antigen. Numbers on the y-axis represent the means of average OD_450_ ± standard deviations (*n* = 3). Statistical significance was determined using a student’s *t*-test.

No clinical signs of disease and no animal died post immunization during the entire experimental period. As indicated in Figure 4, the pattern of serum IgA responses was similar with that of the IgG responses. From 1w to 5w post-immunization, they showed an upward trend, peaked at week 5 and gradually decreased after that. Ducklings in vaccine treated groups showed considerably enhanced antibody levels at week 5–6 post-immunization, and statistically significant differences between these groups and the control groups (*P* < 0.05) were observed. The OD_450_ values of serum IgA and IgG in SL7207 (pVAX-LTB-UL24) (10^11^ CFU) immunized group were 0.472 (Figure 4A) and 1.754 (Figure 4B), respectively, at 5 weeks post-immunization, which were significantly higher than those in the control groups (*P* < 0.05). Significantly higher levels (*P* < 0.01) of anti-DEV IgG and IgA antibodies in the orally immunized groups were observed, compared with the control groups during the entire experiment (Figure 4A and B). Amongst the oral groups, humoral immune responses induced by 10^10^ CFU of SL7207 (pVAX-LTB-UL24) were higher than those induced by 10^10^ CFU of SL7207 (pVAX-UL24), but the antibody levels among these groups showed no difference in significance (*P* > 0.05). In addition, antibody responses of ducklings inoculated with 10^11^ CFU of SL7207 (pVAX-LTB-UL24) were the highest among all the vaccinated groups throughout the entire experiment. In contrast, no specific anti-DEV antibodies were detected in the control groups.

### Mucosal antibody responses of ducklings

**Figure 5:**
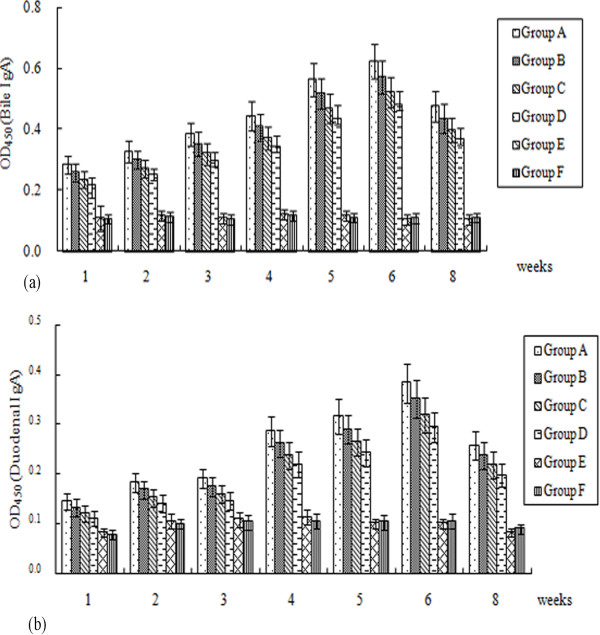
**Mucosal immune responses to DEV measured by ELISA.** (**A**) Bile IgA responses to DEV. (**B**) Duodenum IgA responses to DEV. Bile and duodenum were obtained at different time points and assayed by end-point dilution ELISA using DEV as a coating antigen. Numbers on the y-axis represent the means of average OD_450_ ± standard deviations (*n* = 3). Statistical significance was determined using a student’s *t*-test.

DEV-specific IgA antibodies in the duodenum and bile were measured by ELISA. As shown in Figure 5, the antibody titers generated in vaccine-immunized groups gradually increased and were dramatically higher than those in control groups (*P* < 0.01). At 6 week post-immunization, mucosal IgA antibody titers in duodenum and bile reached their peak levels with the OD450 values of 0.383 (Figure 5B) and 0.623 (Figure 5B) respectively. Moreover, ducklings in groups A, B and C showed stronger mucosal immune responses than those in group D, especially at week 6 post-vaccination, but no difference in significance (*P* > 0.05). The IgA antibody levels of group A were the highest among all the vaccinated groups, with OD450 values ranging from 0.282 to 0.623 in bile and from 0.144 to 0.383 in the duodenum (Figure 5). Notably, the specific bile IgA levels from these groups were significantly higher than those of the duodenum (*P* < 0.01). No antibody induction was observed in the control groups during the experiment.

### Induction of DEV neutralizing antibodies

In SL7207 (pVAX-LTB-UL24) immunized groups, ducklings immunized with 10^11^ CFU SL7207 (pVAX-LTB-UL24) had the highest levels of neutralizing antibody, which indicated that titers of neutralizing antibody were dose-dependent. Detectable titers of neutralizing activity in sera were generated by 10^10^ CFU SL7207 (pVAX-UL24), but they were significantly lower than the titers generated by 10^10^ CFU SL7207 (pVAX-LTB-UL24) (*P* < 0.01). Ducklings immunized with SL7207 (pVAX1) generated considerably lower neutralizing antibody titers, whereas those receiving PBS had no detectable neutralizing antibody responses (Table 1).

**Table 1:** Neutralizing antibody titers in ducklings immunized with DEV DNA vaccines

**Weeks post-immunization**	**Vaccinated groups**				**Control groups**	
	**A**	**B**	**C**	**D**	**E**	**F**
1	1:141.57	1:98.89	1:79.25	1:35.50	-	-
2	1:142.57	1:100.47	1:82.56	1:37.58	-	-
3	1:142.60	1:102.75	1:83.41	1:38.10	-	-
4	1:146.50	1:104.20	1:86.64	1:42.23	-	-
5	1:149.60	1:110.40	1:89.13	1:44.70	1:3.16	-
6	1:148.49	1:108.14	1:89.13	1:44.67	1:3.55	-
8	1:146.80	1:106.00	1:87.40	1:42.64	1:3.27	-

Data represent mean neutralizing antibody titer from 3 serum samples.

-, no neutralizing antibody response was detected.

### Analysis of T lymphocytes in PBL

**Figure 6:**
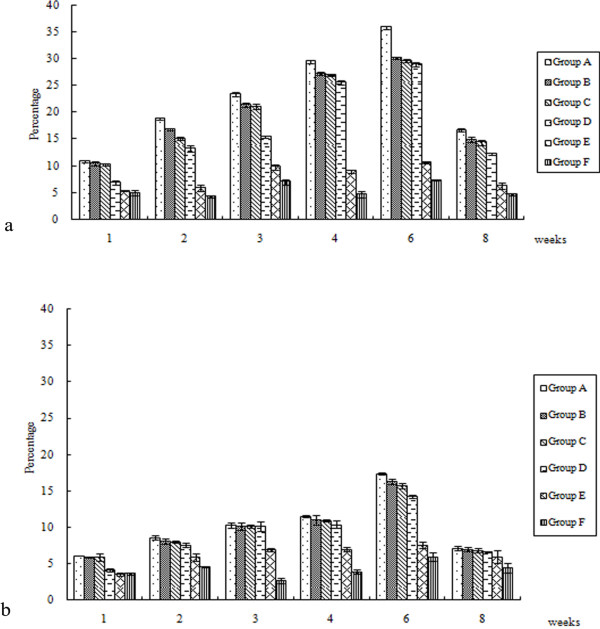
**T lymphocytes in PBL following DEV UL24 DNA vaccination.** 1, 2, 3, 4, 6, 8 weeks after vaccination, the isolated PBL were stained with monoclonal antibodies against duck CD4 (**A**) and CD8 (**B**). The results presented are the mean of all specimens of each group. Statistical significance was determined using a student’s *t*-test.

As shown in Figure 6, the total number of CD4+ and CD8+ cells produced by oral immunization were dramatically higher when compared to the results of control groups (*P* < 0.05) and peak levels were reached between the 4^th^ and 6^th^ weeks.

The strongest induction of CD4+ and CD8+ T cells was observed in the 1 × 10^11^ CFU SL7207 (pVAX-LTB-UL24) vaccinated group at all time points. The CD4+ population of this group was significantly higher than those in other oral groups at 6 weeks post immunization (*P* < 0.05). Compared with the vaccinated groups, the number of CD8+ in 1 × 10^11^ CFU SL7207 (pVAX-LTB-UL24) group was slightly higher but showed no difference in significance (*P* > 0.05) during the entire experiment. The number of CD4+ and CD8+ T cells generated in group B was greater than that in group D, but no significant difference was observed (*P* > 0.05).

### Protection of ducklings against DEV challenge

**Figure 7:**
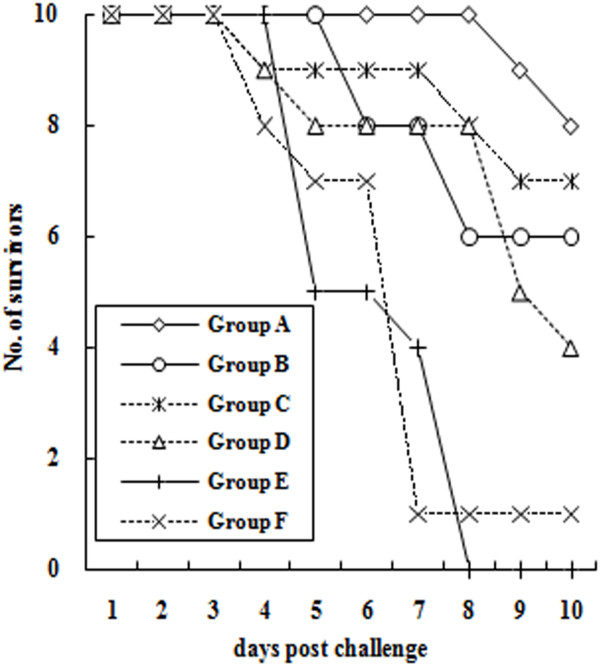
**Numbers of ducklings surviving challenge with 1000 LD**_**50**_**of DEV.** Six weeks after primary immunization, ducklings (10 ducklings per group) were orally challenged with 1000 lethal doses, 50% (LD_50_) of DEV. Mortality was monitored for 10 days after DEV challenge.

As shown in Figure 7, unvaccinated ducklings died quickly from day 4 to 8 and time to death of these ducklings was faster than those of vaccinated ducklings, with statistical significance (*P* < 0.05). The survival ratio (80%) of ducklings immunized with SL7207 (pVAX-LTB-UL24) at doses of 10^11^ was significantly greater than those of ducklings immunized with other vaccines (*P* < 0.05).

## Discussion

DEV has the ability to establish latent infections and an asymptomatic carrier state in waterfowl, which further reinforces the difficulties in the control and prevention of the transmission of DEV [7,27], and may explain why conventional DEV vaccines (inactivated and attenuated DEV preparations) sometimes fail to protect ducks. Therefore, it is urgent to develop a novel or more effective vaccine to control DEV. A DNA vaccine is considered a good choice as it has several advantages, including the simplicity of manufacture, biological stability, cost effectiveness, safety, ease of transport in lyophilized form and the ability to act in the presence of maternal immunity [28]. More importantly, DNA vaccines elicit both humoral and cell-mediated immunity (CMI) stimulated by the presentation of peptide fragments. They have the potential to immunize against a broad range of pathogens, since eukaryotic expression can improve protein folding and therefore enable surface-exposed epitopes to be correctly presented and enable the introduction of post-translational modification [29-32]. These immune responses can be improved by other strategies such as improvements in DNA delivery and the inclusion of adjuvant (either as a gene or co-administered agent) [33]. The evidence supporting this hypothesis comes from this study where we used LTB as a molecular adjuvant and developed a new *Salmonella*-based DNA vaccine delivery system. Ultimately, we demonstrated that DNA vaccines delivered by attenuated *Salmonella* expressing *UL24* gene have a great ability to elicit systemic and mucosal immune responses and LTB from *E. coli* is a very useful adjuvant for DEV DNA vaccines.

As expression of antigens in antigen-presenting cells (APC) by DNA vaccine is a potential means of priming immune responses, targeting DNA to APC is likely to increase DNA vaccine potency [34-36]. In this study, we detected the transcripts of *UL24* and *LTB-UL24* genes by RT-PCR in vivo, indicating that both SL7207 (pVAX-UL24) and SL7207 (pVAX-LTB-UL24) can be expressed by APC (Figure 2). Because of the low number of APC present in muscle, delivery of DNA vaccine through traditional administration (intramuscularly or subcutaneously) would result in poor immune responses [33,37]; while a large fraction of bacteria carrying DNA vaccine are taken up by APC which express and present the antigen after crossing the intestinal mucosal barrier [14]. Therefore we used the oral route to immunize the ducklings. In addition, some component of gram-negative bacteria such as lipopolysaccharide (LPS) has been shown to act as a potent adjuvant which can enhance immune responses [38]. Moreover, attenuated strains of *Salmonella* are attractive candidates for mucosal vaccine vectors, because they elicit both mucosal and systemic immune responses against carried antigens [39,40]. Therefore, attenuated *S.* typhimurium is not only a promising vaccine carrier, but may also act as an adjuvant to stimulate immune responses [37]. Accordingly, the results in this work were consistent with the aforementioned findings. Oral delivery of DEV DNA vaccines was an efficient way of inoculation, achieving high neutralizing antibody titers and ELISA antibodies (Table 1, Figures 4 and 5). Cell-mediated immune responses were significantly stimulated by DNA vaccines delivered by attenuated *S.* typhimurium (Figure 6).

HSV-specific CD4+ and CD8+ T cells are protective in animal models during HSV infection and the antigen-specific CD4+ T cells support later expression of antigen-specific CD8+ T cells [41,42]. In our tests, the number of CD4+ and CD8+ T cells showed an obvious rise in the early stage after immunization with DNA vaccines. A dramatic increase was seen in the CD4+ population, while CD8+ rose to a lesser degree. The most significant induction in CD4+ and CD8+ T cells was observed in 1 × 10^11^ CFU SL7207 (pVAX-LTB-UL24) immunized group at all time points, indicating that SL7207 (pVAX-LTB-UL24) can induce strong cellular immune responses. In addition, CD4+ T cells play important roles in modulating immune responses, up-regulating the costimulatory molecules on APC cells and enhancing their ability to process and present antigen [43]. Therefore the CD4+ T cell response is a prerequisite when using a potential vaccine. Our work also demonstrated that DEV vaccines could provoke both specific CD4+ and CD8+ T cell responses to prevent or control infection.

The cholera toxin (CT) and heat-labile toxin (LT) from *E. coli* are the most commonly used mucosal adjuvants for immunization. Co-administration of CT or LT with antigen can result in substantial enhancement of antigen-specific secretory and systemic antibody responses [19,44,45]. LTB is widely used as an effective adjuvant to induce protective mucosal and systemic immune responses since LTB exhibits low toxicity, which was also demonstrated by our work (Table 1, Figures 4,5 and 6). Amongst the oral groups, SL7207 (pVAX-LTB-UL24) induced obviously better mucosal and systemic immune responses, compared with SL7207 (pVAX-UL24). When comparing the three different doses of SL7207 (pVAX-LTB-UL24), we found that immunogenicity of the DEV DNA vaccine was dose-dependent, with higher doses of SL7207 (pVAX-LTB-UL24) (e.g. 1 × 10^10^ CFU or 1 × 10^11^ CFU) inducing better immune responses. Delivery of the DNA vaccine at a low dose (10^9^ CFU) induced systemic and mucosal immune responses, which were enhanced by increasing the dose of attenuated *Salmonella*. Moreover, ducklings immunized with 10^11^ CFU of SL7207 (pVAX-LTB-UL24) showed the highest anti-DEV mucosal and systemic immune response throughout the experiment.

Challenge studies revealed that higher mucosal and systemic immune responses were more protective against DEV infection. These findings suggest that the DEV DNA vaccine with the LTB gene fused to *UL24* and delivered by attenuated *S.* typhimurium induced protective prophylactic responses against DEV challenge and this DNA vaccine at a high dose (10^11^ CFU) showed a superior protection, demonstrating that LTB is a potential and effective adjuvant of the DEV DNA vaccine.

Taken together, our results indicate that oral immunization with attenuated *S.* typhimurium as a DNA vaccine carrier induces efficient systemic and mucosal immune responses. We report here the first attempt to develop oral DNA vaccines against DEV infection and have shown that orally administered attenuated *S.* typhimurium co-expressing *UL24* and LTB was able to protect ducklings against DEV infection. Consequently, a DNA vaccine encoding the *UL24* gene of DEV and LTB gene of *E. coli* delivered by attenuated *S.* typhimurium may be a promising DEV vaccine.

## Competing interests

The authors declare that they have no competing interests.

## Authors’ contributions

XY carried out most of the experiments and drafted the manuscript. RY, AC, MW and SC critically revised the experiment design and manuscript. JH, BS, DK, XH, ML, ZQ and XY helped with the experiment. QL and YW helped with the addition experiment and revision of this manuscript. All authors read and approved the final manuscript.
